# Addressing Participant Validity in a Small Internet Health Survey (The Restore Study): Protocol and Recommendations for Survey Response Validation

**DOI:** 10.2196/resprot.7655

**Published:** 2018-04-24

**Authors:** James Dewitt, Benjamin Capistrant, Nidhi Kohli, B R Simon Rosser, Darryl Mitteldorf, Enyinnaya Merengwa, William West

**Affiliations:** ^1^ Division of Epidemiology & Community Health School of Public Health University of Minnesota Minneapolis, MN United States; ^2^ School of Social Work Smith College North Hampton, MA United States; ^3^ Department of Educational Psychology University of Minnesota Minneapolis, MN United States; ^4^ Malecare New York, NY United States; ^5^ Department of Family Medicine and Community Health University of Minnesota Minneapolis, MN United States; ^6^ Department of Writing Studies University of Minnesota Minneapolis, MN United States

**Keywords:** fraudulent data, data accuracy, research and design, research activities, data analysis

## Abstract

**Background:**

While deduplication and cross-validation protocols have been recommended for large Web-based studies, protocols for survey response validation of smaller studies have not been published.

**Objective:**

This paper reports the challenges of survey validation inherent in a small Web-based health survey research.

**Methods:**

The subject population was North American, gay and bisexual, prostate cancer survivors, who represent an under-researched, hidden, difficult-to-recruit, minority-within-a-minority population. In 2015-2016, advertising on a large Web-based cancer survivor support network, using email and social media, yielded 478 completed surveys.

**Results:**

Our manual deduplication and cross-validation protocol identified 289 survey submissions (289/478, 60.4%) as likely spam, most stemming from advertising on social media. The basic components of this deduplication and validation protocol are detailed. An unexpected challenge encountered was invalid survey responses *evolving* across the study period. This necessitated the static detection protocol be augmented with a dynamic one.

**Conclusions:**

Five recommendations for validation of Web-based samples, especially with smaller difficult-to-recruit populations, are detailed.

## Introduction

### Misrepresentation in Web-Based Studies as a Challenge to Scientific Validity

Internet-based research is growing in popularity and usability among social science, behavioral science, and health researchers, in part because of the ease and efficiency in recruiting large samples [1,2]. It is also advantageous-to-essential in recruiting niche, hidden, and hard-to-reach populations [3]. A Web-based survey design software has become so user friendly that virtually anyone can design and implement surveys. Web 2.0 survey software such as Survey Monkey, Google Forms, Survey Gizmo, Qualtrics, and others work on a range of electronic devices, are compliant with health regulation for clinical data management, participant privacy and security (eg, Health Insurance Portability and Accountability Act, HIPPA), and offer in-app data analysis and user metrics.

A unique characteristic in Web-based health research is that the researcher(s) typically never meet the participants. This creates a unique challenge to participant validity. As new software technologies have eased implementation from the researchers’ end, a whole new scope of technologies has also developed to complete surveys, fraudulently. In Web 1.0, early research focused on survey item validity to demonstrate that asking health information online could yield truthful responses. In this study, 2 key findings emerged. First, misrepresentation online was not uniform but varied across health foci [4], and for socially sensitive items, computerized surveys yielded higher (interpreted as more truthful) response rates [5]. The first case studies of multiple participation by a single person were reported [6], followed by studies reporting participation by ineligible individuals posing as eligible subjects [7]. The evolution of user-friendly software created not only Web 2.0 but also the capability for spamming technologies and approaches that create new risks to Web-based research validity.

Wikipedia defines spam as *unsolicited or undesired electronic messages*. Applied to social science and health research, “spam” is used as a catch-all term referring to sets of invalid or fraudulent data responses. Web-based survey spam has been categorized with the assumption that the source of spam comes from an individual person or group of people who, for a variety of reasons (eg, to earn compensation, to politically influence survey findings, or out of interest), would complete a Web-based survey multiple times [8]. In response, protocols to verify the validity and uniqueness of each survey are considered essential in distance studies [7]. Deduplication is the process used to confirm that each survey is from a unique person, whereas cross-validation comprises internal validity checks to ensure the data are consistent and interpretable across the study. The major challenge in both deduplication and cross-validation is detection: how to ensure such protocols can distinguish valid from invalid data [7]. As spamming has evolved to become a greater threat to Web-based research integrity, deduplication and cross-validation protocols need to be more sophisticated.

### Data Validation Protocols

Most data validation protocols rely on a 2-part algorithm consisting of partially automated validation checks and manual validation checks [7]. Manual, automated, or hybrid manual-automated protocols use combinations of data to verify the survey responses (internal validity) while ensuring non-duplication. Typical analytic data used include tracking all attempts made on study entry (including documenting repeated attempts at study entry by changing answers to screening information), internet protocol (IP) address, a timestamp of the survey start and end date, duration of the survey, and completion status. Examples of issues that have emerged in regard to using survey metadata to determine eligibility are IP masking and data-generating software. Tran et al [9] describe the functioning of the software, explaining that users can generate a complete dataset, each assigned with a unique ID and easily downloadable in a comma-separated value file. Spammers can easily obtain this kind of software as well. One of the first results in a cursory Google search for “software to auto fill an online survey” pulls up “Coby 2.0,” a Bot program that advertises undetectable, human-like data, with unique virtual private network (VPN), live panel statistics, an automated info generator, and fake email generator (registers the emails with a host site to pass verification), and Captcha verification software, with instructions on how to run this software on a Web-based survey. The implication for social science behavior and health researchers is that spam itself is evolving, shifting from being manually created by individuals to automated by software. This development introduces new threats to Web-based research validity and challenges to the survey validation process.

In internet-based social science, behavioral science, and health research, there is little published information in the area of data validation and fraud. All the case studies we could find refer to protocols designed to handle large survey populations. Take, for example, research on HIV prevention on gay, bisexual, and other men who have sex with men (GBM), an area of research where Web-based recruitment and distance surveys have become popular. At least 3 Web-based studies have published data deduplication and cross-validation protocols: the *Wyoming Rural AIDS Prevention Project* (*WRAPP*) [10], total attempts N=1900; the *American Men’s Internet Survey* (*AMIS*) [11], total attempts N=14,899; and the *Sexually Explicit Media* or *SEM study* [7], total attempts N=1254 surveys. All 3 studies published data validation protocols using some mix of automated detection to flag “suspicious” surveys, followed by manual confirmation to confirm duplicate and/or invalid surveys.

Recruiting smaller “niche” populations in Web-based research introduces special challenges to validity. When the population is small, it may not be cost-effective to computerize validation checks. Smaller samples are typical when a behavior or health concern is rare or novel, 2 situations where typically less is known about the phenomenon. This makes it difficult to detect and verify atypical cases. In such a situation, researchers may need to institute a manual data validation protocol with multiple external validation checks to ensure high-quality data. Using the *Restore* study as a case study, and comparing it with the current standards in the field, this paper addresses the issue of detecting Web-based survey spam, detailing an appropriate protocol necessary to identify suspect surveys in this small-sample study. This paper has 3 aims: (1) to outline the basic components of a data validation protocol for smaller samples, (2) to identify challenges encountered and solutions tried to address these challenges, and (3) to make recommendations for future research.

### Restore: Study Description

The *Restore* study was a Web-based study of GBM treated for prostate cancer completed in 2015-2016. The study focus was the effects of treatment to inform development of a Web-based rehabilitation curriculum tailored to the population. Only the second prostate cancer in GBM study to be funded by the National Institutes of Health, *Restore* had the challenge to recruit for a rare event (as prostate cancer is a disease of older men) within a sexual minority. The challenge in recruiting a “minority within a minority” is illustrated by the sample sizes in the extant literature. At the start of the study, only 4 quantitative studies of GBM prostate cancer survivors had been published and a fifth doctoral thesis abstracted, with GBM sample sizes of 12 [12], 15 [13], 92 [14,15], 96 [16,17], and 111 [18], respectively. Given GBM represents a geographically diverse population, internet recruitment and Web-based surveys have become the standard methods in GBM health research. Sexual minorities were early adopters of new technology [19] and remain disproportionately likely to be online. GBM comprise a vibrant virtual community whose uses of the internet and apps include dating and sex-seeking online, community building, sharing information, and accessing goods and services [20].

Studies of GBM prostate cancer survivors are rare, in part because of challenges in recruiting this demographic. Prostate cancer registries typically do not collect data on sexual orientation, leaving this population invisible to clinical research. Except for a few cities with large geographic concentrations of GBM, most cities lack sufficient numbers of GBM seeking prostate cancer treatment at any one time to make in-person support groups, viable. Sexual minority status [21], prostate cancer [22], and the sexual and urinary effects of treatment [23] are all potentially stigmatizing, creating further psychological barriers to participation. Unlike HIV, some survivors of prostate cancer may no longer identify as such after successful treatment. Thus, for multiple reasons, GBM prostate cancer survivors represent a hidden, difficult-to-reach population who may only be accessible in significant numbers through the internet [24,25]. Given the social isolation and dual stigma—GBM with prostate cancer experience—Web-based support groups have become an important, vital place for these men to have access to counseling, support, and care.

Generating a sample from a virtual community has specific challenges. Without a precise enumeration of the population, it is difficult-to-impossible to establish the sampling frame from which to sample. Web-based surveys face additional recruitment challenges as it is often more difficult to restrict access to a Web-based survey and to guarantee the validity of the population and consequent quality of the data. This was true in the case of the *Restore* study when, not long after advertising the Web-based survey on social media, it became subject to a spam attack.

Another significant challenge in the case of the *Restore* study was the need to balance survey protection with accessibility. Prostate cancer largely impacts an older demographic (mean age of this sample was 63.42 years, standard deviation, SD, 8.19). So, the survey needed to be designed for participants who were less technology-savvy. Some of the survey design solutions that were considered and could be useful in other circumstances presented potential obstacles to recruiting the study’s population. For example, requiring participants to first contact the study to obtain a unique ID, password, or individualized link to the study could both protect a Web-based survey while externally validating the population. This option can and should be considered when a researcher or organization sponsoring the research has an existing relationship with the target group. In case of the *Restore* study, such a relationship was more tenuous, with most of the prostate cancer survivors having no explicit relationship with the university-based researchers conducting the study. In addition, although these men were active online, it was unclear what level of internet familiarity they possessed, and so, it was important to make the process of recruitment and enrollment as welcoming, easy, and streamlined as possible.

## Methods

### Study Design

In many ways, the *Restore* study reflects a typical formative research study in the social sciences, behavioral, and/or health fields. The study design involved a formative qualitative research phase (listening to GBM’s experience of prostate cancer in individual interviews) and a measurement development phase (to measure treatment outcomes specific to the target population). The third aim, to conduct a cross-sectional, Web-based, quantitative survey assessing GBM prostate cancer survivor’s sexual functioning and rehabilitation needs following treatment, is the focus of this report. The core components of the Web-based survey included the following domains: eligibility screener, consent process, demographic questionnaire, sexual identification, prostate cancer treatment history, measures of sexual functioning, an HIV/ sexually transmitted infection status and risk inventory, a section on primary relationships, measures of physical and mental health, alcohol and tobacco use, and a tailored needs assessment of what GBM with prostate cancer want in rehabilitation [26].

### Recruitment/Enrollment

The study was launched on October 21, 2015, and ended on January 1, 2016 (72 days). Given the difficulties identifying and recruiting GBM with prostate cancer into studies, a methods goal was to establish the utility of using Web-based methods to recruit this population. The primary focus of recruitment was through a community partner’s email listserv. *Malecare* is a nonprofit organization providing support and advocacy for survivors of cancer who agreed to send an email invitation to their members. Email invitations were sent seeking to recruit men treated for prostate cancer, residing in the United States and Canada, aged 18 years or older, and who identified as GBM (by self-report). (Given prostate cancer is a disease of older adults, the age criterion was a technicality, reflecting the minimum legal age to consent to research, and was not anticipated to restrict the study). After much discussion, a link to the survey, noting a US $50 gift card as compensation, was advertised on the social media site, Facebook. This allowed the advertisement to be “shared” by other public pages of prostate cancer community organizations.

To complete the survey, participants first completed a screening tool verifying eligibility and then a Web-based questionnaire, which took about 45 to 60 min to complete. For an additional US $10 incentive, participants could refer their partners, friends, and family to take a companion survey on caregiving and social support. Consent for this low-risk study warned participants that some questions were sexually explicit and potentially embarrassing. All research was undertaken under the oversight of the University’s human subjects’ protection program.

### Data Validation

Initial validation was confirmed at study entry by completion of the screener. This required all enrollees to click through (from the email sent out by *Malecare*), confirm each eligibility criterion by checking 4 boxes (≥18 years, residing in the United States or Canada, and identifying as a GBM who has completed treatment for prostate cancer), and provide consent (by clicking on multiple screens that they had understood key aspects of the survey and wished to participate). A summary page required them to verify again that they met all the eligibility criteria, and to confirm they had not completed the survey previously.

Initial survey protection features included “Prevent Ballot Box Stuffing,” a feature of Qualtrics that stores a cookie on the user’s browser, and “Prevent Indexing,” a feature that prevents the survey from being indexed by search engines.

Our manual protocol for data validation was adapted from our prior large studies on GBM (using automated and hybrid protocols), utilizing the standards summarized by Baker and Downes-LeGuin [8] who identify 8 indicators of suspect survey entries (see Textbox 1). Similar to Baker and Downes-LeGuin’s “3 strikes and you’re out” rule, no 1 indicator was deemed sufficient to call a survey invalid. Rather, indicators were used to “flag” survey entries as suspicious to study staff.

**Figure 1:**
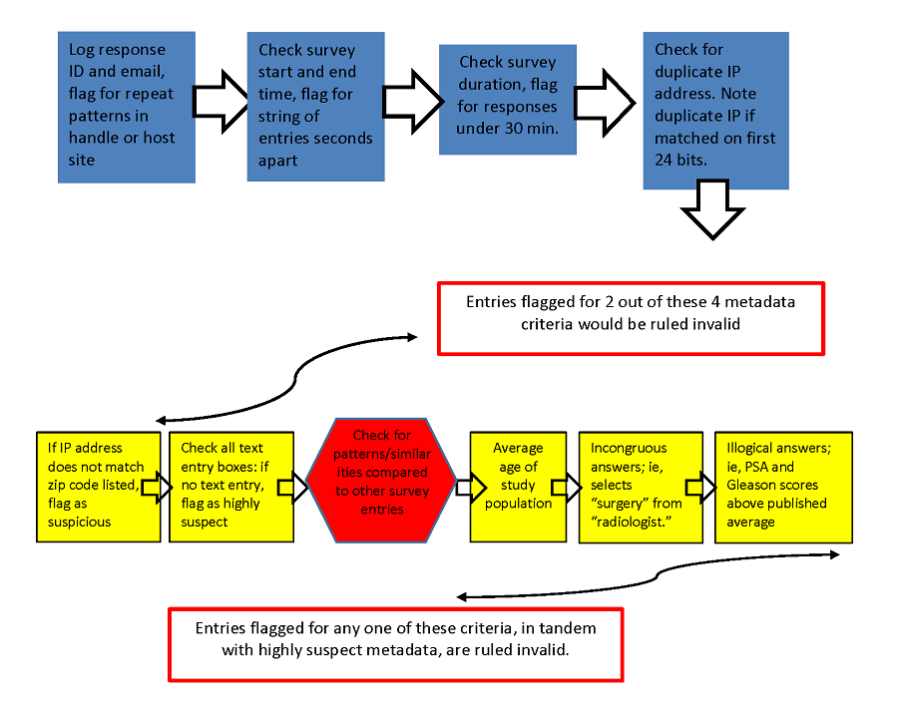
Restore study data validation protocol. ID: identification; IP: Internet Protocol; PSA: Prostate-Specific Antigen Test.

In addition, internal checks of survey metadata were conducted against the participant log and survey responses (see Figure 1). Submissions were flagged automatically for incongruity between these items and then checked manually by study staff and independently by the principal investigator. In the case when suspicious responses to medical questions arose, responses were flagged and sent to the team’s prostate cancer medical expert to confirm if the response pattern was clinically impossible and/or statistically highly improbable.

For all analyses, duplicate and/or invalid submissions were treated as 1 invalid group. A priori, only complete survey submissions were to be included in data analysis. Thus, any incomplete survey submissions were to be assigned as invalid.

**Baker and Downes-Le Guin’s 8 characteristics of suspicious surveys.**

Unusually short completion times compared with the median interview length

Selection of all items in a multiple response or other obvious cheating behavior in qualifying questions

Selection of bogus or low probability answers

Internal inconsistencies

Low differentiation or “straight lining” in grids

High levels of item nonresponse

Failure of verification items in grids

Gibberish or duplicated responses in text entry boxes

## Results

### Participants

**Figure 2:**
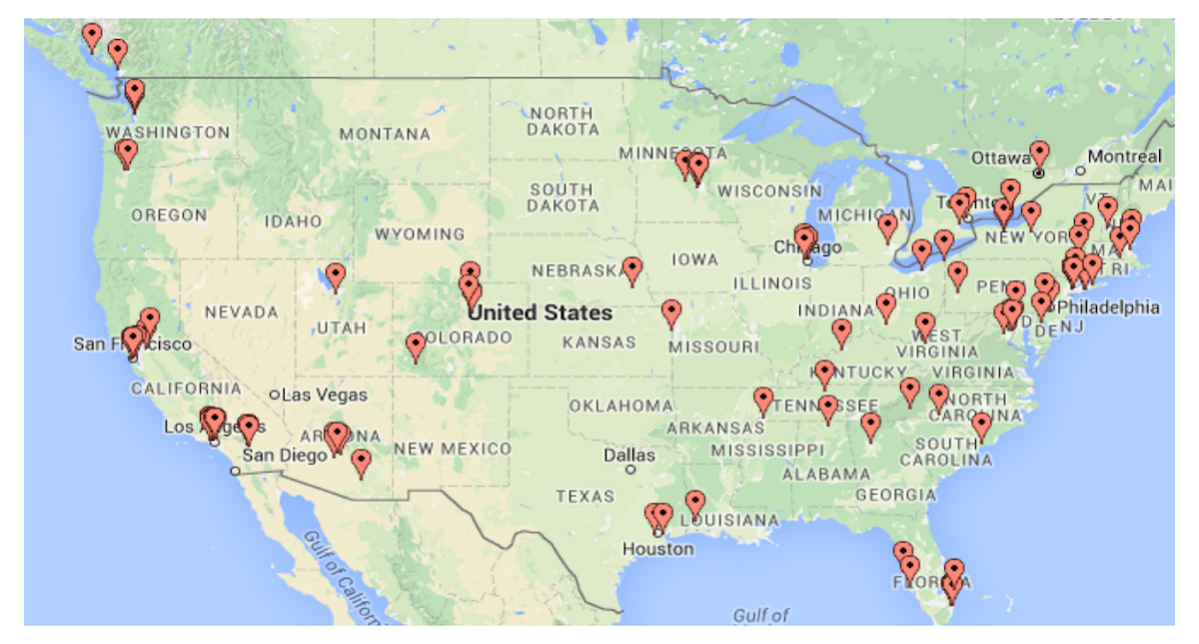
Geographic distribution of the sample (N=193 gay, bisexual, and other men who have sex with men [GBM] prostate cancer survivors).

Participants’ characteristics are summarized in Multimedia Appendix 1. To summarize, the typical participant in this study is a white, non-Hispanic, well-educated male, in his 60s, living in the United States, and self-identified as gay. Geographically, the sample appears diverse living across North America as validated by a residence zip code (see Figure 2), and cross-validated by where they sought treatment. Medically, 69.4% (134/193) reported their cancer as having been successfully treated, the remainder reporting either still undergoing treatment or reporting their cancer has progressed. Against the stereotype that older participants are not online, most participants reported being online 20 or more hours per week and most reported using multiple platforms to access the internet.

### Evidence of a Spam Attack

**Figure 3:**
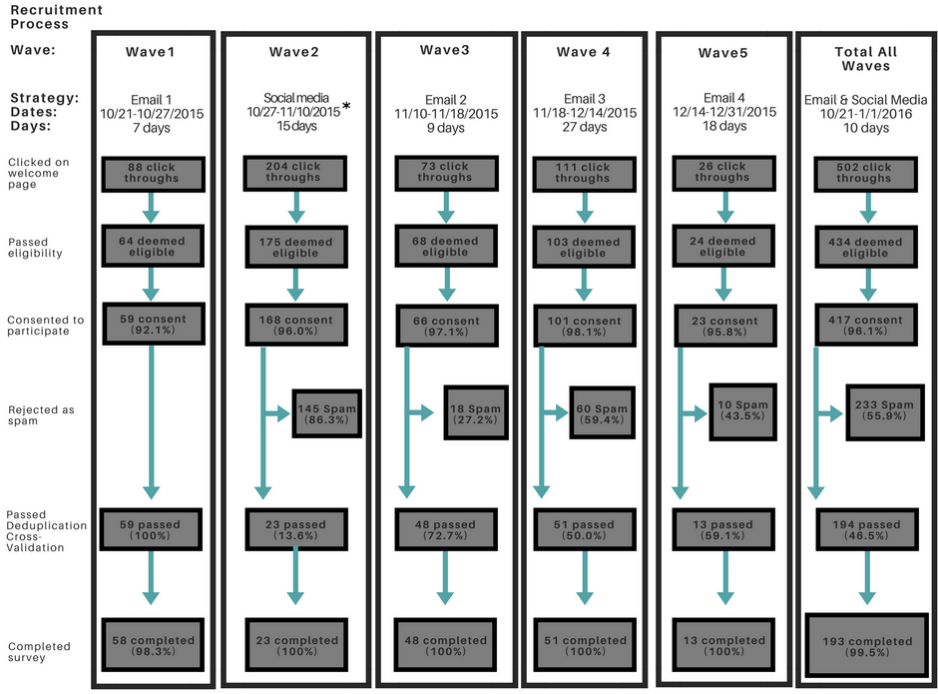
Recruitment process and participation rates in the Restore study. All advertising on social media removed on 11/5, but survey link left open to keep spammers on the site while members sent to new link.

A spam attack on the survey began in the second recruitment blast (Wave 2), 2 days after US $50 online gift certificate compensation was advertised on Facebook (see Figure 3). Study staff noticed a sudden increase in survey attempts, many of which at first seemed potentially valid. Ultimately, staff rejected each survey as suspect for multiple reasons, based on survey metadata and survey responses. A total of 289 survey submissions, representing 60.4% (289/478) of submitted surveys, were ultimately rejected as likely spam.

Metadata issues that suggested a common source were flagged as suspicious and ultimately rejected as spam. Criteria included short response time (under 30 min), IP addresses that did not match the zip code provided by respondent, duplicate IP address, an email address that followed an unusually predictable convention in the handle, and all were from the same host site (eg, abc123@.me.com). Initially, survey entries that were flagged followed a consistent pattern. First, these surveys provided a highly improbable age (18-35 years) at which to receive a diagnosis of prostate cancer. (Less than 1 in 10,000 men under age of 40 years is diagnosed with prostate cancer). The data on prostate cancer specific items were either “forgotten” or also statistically less likely (>20 for prostate-specific antigen or PSA tests and >6 for Gleason). The information on treatment combined statistically improbable-to-impossible treatment regimens (eg, radical prostatectomy, then watchful surveillance then radiation treatment, then diet). In addition, there were mismatches of treatments experienced with the providers seen (eg, radiation from an urologist or surgery from an oncologist). On the discrimination experiences scale, reports of serious discrimination experienced in hospital were common but based on unusual attributes (eg, weight, height, and appearance discrimination but not race or sexual orientation). In terms of rehabilitation, the surveys reported rare treatments that would be highly unlikely if the patient had not disclosed their sexual orientation and behavior to their provider (eg, recommended use of dilators and dildos, and butt plugs to a man who had not disclosed his sexual orientation, or to a man who stated he exclusively engaged in insertive sex). Consistent with Baker and Downes-LeGuin (2007), suspicious surveys consistently left text entry boxes for qualitative responses blank. None of these items by itself would rule out a survey as invalid, rather each participant’s answers was read as a case study to see if the overall profile was credible. This meant the consistent combination of multiple statistically or medically unlikely response items would flag them as suspect, and ultimately, the combination of multiple items or profile as suspect would result in them being rejected as invalid.

Over time, the answer patterns began to shift making detection of spam more difficult. Although completing time remained suspicious, some survey demographics were within a probable range, whereas other data stayed improbable. For example, age at diagnosis and PSA and Gleason scores all were within the plausible range of responses. This shift in how invalid responding was coming in was significant enough to require adaptations to the study’s data validation and deduplication protocol. First, the invalid entries were able to “pass” through some aspects of the protocol (eg, entries had non-duplicate IP and valid zip code, respondent passed reCAPTCHA verification). Next, the invalid entries to some of the medical and behavioral questions shifted toward becoming more plausible. Fortunately, other responses in the suspicious surveys still followed a convention in the metadata that identified them as suspicious. Key suspicious data entries included repetitious email address at the same host site, entries that began seconds after a previous submission had ended, and IP addresses that did not match the zip codes listed in the demographic questionnaire. Across 1 set of spam, a consistent mistake was observed on 1 datum involving a misinterpretation of how to state a date of birth. Across others, suspicious surveys tended to be completed in the early hours of the morning. These were only identifiable by checking the process analytics, manually reading each whole survey interpreting all data in relationship to each other, and reviewing each survey’s demographics (eg, start and end completion times, style of email address provided) in relationship to other surveys.

After 21 days of reviewing the spam entries on the survey, the flow of spam was diverted. We kept the old link open while launching a copy of the survey with a stricter recruitment protocol (restricted to email invitations and no social media, with more validation steps between the screener, consent, and survey). This new link was only advertised through the community partner’s email listserv and was never spammed. Within a very short period (2-3 days), the original link only received spam, whereas the restricted new link only received valid surveys.

No compensation was paid to surveys that were identified as spam. When requests for payment were received from respondents whose surveys were identified as invalid/spam, they were informed there was a question about some of their data and asked to call on the 1-800 study line to leave a call back number to verify their answers. This was sent to all the spam surveys, but no one followed through with this request.

## Discussion

### Detecting Fraudulent Surveys

Ensuring the quality of data in Web-based surveys is essential to maintain the credibility of Web-based research. In situations where external validation is not possible or appropriate, a rigorous deduplication and cross-validation protocol can provide researchers with confidence in their data. We highlight that the majority of surveys received in *Restore* were rejected as spam. Clearly, even in small-sample studies, fraudulent participation is a serious threat.

Detecting fraudulent responses in Web-based surveys is a rapidly evolving challenge. To ensure the highest data quality from Web-based surveys, researchers need to adapt existing data validation protocols and publish their experience in detecting spam. In this case study, although some of the spam attack may have been completed in-person by a human being, it is also possible the invalid entries on the survey were automatically generated by software. Both explanations would account for how the spamming evolved across the study.

There are 4 main lessons learned from this case study. First, the identification and rejection of 289 (60.4%) surveys as spam reinforces the need for similar Web-based studies to monitor the threat of spam. Studies without such protocols should be viewed as methodologically weak, potentially not meeting minimum standards for publishable research. Best practices to monitor and reduce the danger of spam include keeping the recruitment period short and, given the experience of the *Restore* study, avoidance of advertising on publically accessible social media sites (wherever possible). Second, the standard to develop a written deduplication and cross-validation protocol for the duration of a study, which was previously identified as a “best practice,” now needs to be updated. Given how the spam responses evolved across this study, instead of a static protocol, written before the start of recruitment and implemented consistently across a study, researchers need dynamic protocols that can evolve in response to evolving spam attacks. To ensure consistency over the study period, “evolving protocols” may need to vary rules as the study proceeds and necessitate a retrospective analysis phase that applies uniform standards after the recruitment period has ended. Moreover, this reinforces the essential element of having manual checks by multiple staff to detect subtle shifts in responding patterns. Third, although a common practice in validation is to assign human-like attributes to the spammer (ie, giving a name, character, or identity to the spammer), the risk of spam from automatically software-generated programs is an emerging threat. To the extent that assigning human-like attributes reinforces perceiving the attack as human, consistent, and static, it should be avoided. Instead, to remove spam, even in small-sample size studies, a mix of manual inspection and automated review to flag suspicious entries is needed. Fourth, when spam attacks occur, researchers should consider a novel solution piloted in the *Restore* study. Maintaining an old link to collect spam, while opening an alternative more restricted link for members, enabled the *Restore* study to quickly recover from the spam attack and complete the study.

### Considerations in Designing Web-Based Survey Validation Protocols

In this case study, we encountered several design considerations with implications from validating survey samples. First, like many recruitment studies using email invitations, the *Restore* study sent out invitations to a large listserve, resulting in rapid initial recruitment. Given the volume of response, it was initially challenging to carefully process each survey. An alternative approach would be to have a gradual recruitment roll out. This would have allowed staff more time to gain experience in detecting suspicious surveys. But it is a trade-off, as it also allows more time and opportunity for spammers to gain experience in spamming the study. Second, detailed record keeping of all incomplete entries throughout every stage of the survey, especially those who did not complete the screener and consent, could have helped early identification of spam patterns.

Third, all the published protocols in the field to date, to the best of our review, report using a static standard or rigorous protocol to categorize an entry as “valid” or “invalid.” Although this is consistent with rigorous best practices, in the case of the *Restore* study, it proved insufficient. Dynamic protocols designed to adapt or evolve will be more responsive, but inherently vulnerable to behavioral drift. Fourth, with permission from the institutional review board (IRB), including some form of retrospective external validation (such as a phone call, Skype contact, or other personal validation) proved useful both to confirm the survey as suspicious and to provide a process where, in the case of a survey incorrectly rejected, a participant could confirm its validity. Although for large studies this may introduce feasibility concerns, for smaller studies, building retrospective external validation into the protocol may minimize fraudulent attacks.

Finally, if most spamming was motivated to earn compensation, does removing compensation solve the threat of spamming? We think not. Although compensation may increase the threat of spam, in a recent study, spamming by those not seeking compensation was actually higher than by those seeking compensation [7]. Discussions with colleagues who have conducted Web-based health surveys without compensation have revealed similar concerns about spamming (undertaken for political gain). Ethically, the US $50 compensation in this study reflects the US research practice to compensate for time, effort, and other costs. It was chosen to be sufficient to compensate for time, computer use, and effort, but not be so great as to incentivize or induce respondents to participate.

### Comparisons With Prior Literature

There are 2 examples of best practices in the field of social and behavioral research: the American Men’s Internet Survey and the Wyoming Rural AIDS Prevention Project. The main similarities here are the study population and time frame— *Restore*, AMIS, and WRAPP all used Web-based research methods to study GBM in the past decade. All 3 studies have published data validation protocols and cross-validation strategies based off of automated validation checks. Distinctively, AMIS conducted one of the largest studies of online GBM to date, with minimal occurrence of invalid entries. The goal of this study was to collect 10,000 surveys annually to monitor behavior patterns among GBM internet users. Of 14,899 total “eligible” (ie, adult GBM) participants screened, only 709 (4.76%) surveys were determined to be from duplicate participants. In the case of the WRAPP study, a Web-based HIV intervention evaluation project targeting rural GBM, a similar spam pattern to that of *Restore* occurred. Of 1900 submissions, 627 (33%) were considered to be invalid.

Strength of the AMIS protocol includes its use of both metadata and survey response flagging to determine eligibility, noting automated and manual data of the respondent (IP address, completion rate, demographic characteristics, GBM status, etc). The AMIS protocol does not attempt to categorize or source ineligible participants with any particular quality, but simply refers to valid/complete/nonduplicate entries as “successful” and all others as “unsuccessful.” A limitation of the AMIS protocol would be framing the overall method as “deduplication,” and when based on more current findings, it can be deduced that some spam can now look unique/nonduplicate but still be invalid.

The WRAPP protocol’s strength is also in its 2-part approach, using both survey responses and internal metadata (such as IP address, Web browser information, user-determined variables such as username, password, and email, as well as optional user-provided variables such as phone number). Furthermore, they report invalid data broken down into 4 categories: “Infrequent, Persistent, Very persistent, and Hacker” [10]. Future research in this area should focus on how to present invalid data and possibly even comparison of invalid data within categories.

Both studies represent state-of-practice data validation researcher. The *Restore* study builds on these in 2 significant ways: first, by tailoring such research to small sample studies, and second, by testing a dynamic protocol.

### Recommendations for Future Research in Small Web-Based Studies

We recommend researchers consider 6 recommendations when designing small Web-based samples.

#### Use a Formal, Written Deduplication and Cross-Validation Protocol

Given the number of studies reporting suspicious surveys, and the percent of surveys identified as spam, validation of each survey as a unique contribution from an eligible participants is essential for all Web-based survey research. We encourage investigators, reviewers, IRBs, and everyone involved in Web-based research to promote survey validation as a minimum standard of rigor. Studies where external validation of participants is not possible or appropriate, there is a need for some kind of survey deduplication and cross-validation process. Else, they should be rejected as below minimal scientific standards. To aid the field, researchers should make their protocols available, either by publishing them as a case study as done here or as Web-based appendices to the main paper reporting the results. Because each study is unique, existing protocols will need to be tailored to the target population and adapted to best fit the needs of the study.

#### Adopt a Rigorous Authentication Protocol While Carefully Considering Who May Be Excluded

Designing a deduplication and cross-validation protocol involves complex tradeoffs, balancing authentication against both subject burden and potential exclusion. Overly simplistic protocols—for example, checking name or IP address duplicates—are weak and too easily circumvented. Rigor may also exclude some valid participants. For example, in our study, some male couples might live together, share the same IP address, and both be diagnosed with prostate cancer. In another study, skip patterns might lead to short completion times. When rejecting a survey, we recommend encouraging anyone who thinks they have been “excluded in error” to contact the study directly.

#### Decide Upon an Automated, Manual, or Automanual Hybrid Validation Approach

When establishing a survey deduplication and cross-validation protocol, consider the cost versus the benefit of automated and manual validation checks. Although a combination is likely optimal, for small-sample Web-based studies, automated validation may not be cost-effective or possible. For manual checks, especially in the first weeks of a study, multiple people independently assessing validity is preferred. Multiple people have at least 2 advantages. First, different staff might likely develop different strategies to detect fraudulent patterns, whereas some may not pick up on some patterns at all. By using a team approach, staff can act as reliability checks on each other, ultimately helping to ensure that a rigorous validation is achieved. Second, in the situation where spam evolves, multiple staff monitoring validity is more likely to recognize subtle changes than 1 person working alone.

#### Consider External Validation Checks Either Prospectively or Retrospectively

Any study, regardless of sample size, should consider adding an external validation process in Web-based surveys. Similar to in-person study protocols where researchers confirm a person’s identity, Web-based studies should consider whether a prospective external validation check (eg, a phone or video call to the study) is needed. In studies on sensitive topics, with stigmatized populations, or with hard-to-reach populations (all of which we experienced in the *Restore* study), researchers need to recognize a trade-off. Although external validation may be optimal, it also can introduce a sizable (possibly even insurmountable) barrier to participation. Before requiring external validation checks, acceptability studies and consultation with the community (eg, through community advisory boards) would appear prudent to avoid overly restrictive and intrusive validation procedures with potential to defeat the aims of the study. An alternative approach, which we piloted in this trial, is to state as part of the consent process, that the researchers may require a phone call to the study before the payment if researchers have questions about the survey responses. This is especially useful for research restricted to a geographical area (eg, *Restore* was restricted to North America), as it creates an additional deterrent to fraudulent participation from outside that geographic region.

#### Rapid Recruitment May Be Preferable to Rolling Recruitment

A key observation from the *Restore* study was that invalid entries evolved over time approximating valid responses. Although we cannot know whether this was human learning or automated shaped responses, a rapid recruitment protocol prevents spammers and programs from becoming savvy to the subject area and limits the time frame for evolution of responses to occur.

#### For Small Datasets, a Written Protocol Can Only Go So Far

Although data-validation protocols are the gold standard, for novel areas of research and areas where little is known about the population, valid responses can be hard to predict. For highly innovative research, protocols need to be flexible enough to allow for adaptability. In such cases, starting with an initial “base” protocol and then evolving the protocol with close monitoring (as was done here) may be necessary. Then, reading each survey as a whole to understand how the story fits (or fails to fit) together is crucial to assessing its internal validity.

### Conclusions

In summary, spam on internet-based health surveys appears common, pernicious, and detrimental to Web-based research in the social sciences, behavior, and health disciplines. The threat of spam requires complex and comprehensive solutions to confirm and validate survey responses. Spam appears to be changing in significant ways, including from individual spammers generating multiple, falsified survey responses to spammers using software to autogenerate smarter fraudulent data. Updated protocols that address these advances in technology, and are tailored to the size and nature of the population, are necessary to address these threats.

## Appendix

Multimedia Appendix 1 Demographic, sexual, and medical characteristics of study participants (N=193 gay, bisexual, and other men who have sex with men treated for prostate cancer).

## Abbreviations

AMIS: American Men’s Internet Survey
GBM: gay, bisexual, and other men who have sex with men
IP: Internet protocol
IRB: institutional review board
PSA: prostate-specific antigen
WRAPP: Wyoming Rural AIDS Prevention Project
