# Supplementary material for: Addressing Participant Validity in a Small Internet Health Survey (The Restore Study): Protocol and Recommendations for Survey Response Validation
Source: JMIR Res Protoc. 2018 Apr 24;7(4):e96. doi: 10.2196/resprot.7655 (PMC5941092; doi:10.2196/resprot.7655)
Supplement: Multimedia Appendix 1 [file resprot_v7i4e96_app1.pdf]

Table 1.

Demographic, sexual, and medical characteristics of study participants (N=193 gay, bisexual, and other men who have sex with men treated for prostate cancer).

[a]Excludes “don’t remember/don’t know” and “refuse to answer”

Characteristic		n (%)
**Nationality**		
	Canada	10 (5.2)
	United States	183 (94.8)
**Gender**		
	Male	192 (99.5)
	Transgender, male to female	1 (0.5)
**Age (in years)**		
	40-49	9 (4.7)
	50-59	55 (28.5)
	60-69	82 (42.5)
	70-79	43 (22.3)
	80-89	4 (2.1)
**Race**		
	White	172 (89.1)
	Black/African American	9 (4.7)
	Asian American	4 (2.1)
	American Indian /Alaska Native American	2 (1.0)
	Native Hawaiian/Pacific Islander	1 (0.5)
	Other	5 (2.6)
**Ethnicity**		
	No Hispanic origin	186 (96.4)
	Hispanic	6 (3.1)
**Education level**		
	Less than high	1 (0.5)

	school	
	High school or General Education Development GED	6 (3.1)
	Some college or associate's degree	37 (19.2)
	Bachelor's degree	69 (35.8)
	Graduate degree	80 (41.5)
**Sexual orientation identity**		
	Gay/homosexual	175 (90.7)
	Bisexual, other	18 (9.3)
**Sexual orientation outness**		
	Not out at all	4 (2.1)
	Out to a few people	30 (15.5)
	Out to about half the people I know	9 (4.7)
	Out to most people	33 (17.1)
	Out to all or almost all people I know	117 (60.6)
**Current relationship status (to a man)**		
	Single	58 (30.1)
	Dating	13 (6.7)
	Married or in a long-term relationship	103 (53.4)
	Widowed, divorced, no longer in relationship	14 (7.3)
**HIV status**		
	HIV-negative	168 (87.0)
	HIV-untested or uncertain	1 (0.5)
	HIV-positive (infected before treatment for prostate cancer)	21 (10.9)
	HIV-positive (infected since	3 (1.6)

	treatment for prostate cancer)		
**Gleason score at diagnosis**			
	2-5	30 (15.5)	
	6	48 (24.9)	
	7	61 (31.6)	
	8	16 (8.3)	
	9	11 (5.7)	
	10	2 (1.0)	
	Don't know/don't remember	31 (16.1)	
**Treatment (medical castration)**			
	Prostatectomy only	99 (51.3)	
	External radiation and/or brachytherapy	35 (18.1)	
	Systemic (surgery and external radiation with or without	54 (28.0)	
	Diet or alternative therapy (eg, selenium, vitamin E)	4 (2.1)	
	Watchful surveillance	1 (0.5)	
**Current status (check all that apply)**			
	Still in treatment	34 (17.6)	
	Finished treatment and cancer is undetectable	134 (69.4)	
	Still have detectable levels of prostate cancer	23 (11.9)	
	My prostate cancer has progressed	12 (6.2)	
**Clinical characteristics at diagnosis**			
	Time since diagnosis (years)	186 n[a]	5.7 (4.5) Mean (SD)

	PSA level	149 n[a]	7.6 (6.5) Mean (SD)
	Gleason score	161 n[a]	6.5 (1.5) Mean (SD)
**Internet use per week**			
	Never	0	-
	Less than 1 hour per week	1	0.5%
	1-5 hours per week	11	5.7%
	5-20 hours per week	70	36.3%
	More than 20 hours per week	109	56.5%
**Devices used to access Internet**			
	Desktop computer	118	61.1%
	Laptop computer	141	73.1 %
	Tablet (eg, iPad, Nook, Kindle)	104	53.9%
	Phone (eg, iOS, Android)	138	71.5%
